# Interaction between Mu and Delta Opioid Receptor Agonists in an Assay of Capsaicin-Induced Thermal Allodynia in Rhesus Monkeys

**DOI:** 10.1155/2012/867067

**Published:** 2012-05-14

**Authors:** S. Stevens Negus, Ember M. Morrissey, John E. Folk, Kenner C. Rice

**Affiliations:** ^1^Department of Pharmacology and Toxicology, Virginia Commonwealth University, P.O. Box 980613, Richmond, VA 23298, USA; ^2^Chemical Biology Research Branch, National Institute on Drug Abuse and National Institute on Alcohol Abuse and Alcoholism National Institutes of Health, DHHS, Bethesda, MD 20892, USA

## Abstract

Delta opioid agonists enhance antinociceptive effects of mu-opioid agonists in many preclinical assays of acute nociception, but delta/mu interactions in preclinical models of inflammation-associated pain have not been examined. This study examined interactions between the delta agonist SNC80 [(+)-4-[(**α**R)-**α**-((2S,5R)-4-allyl-2,5-dimethyl-1-piperazinyl)-3-methoxybenzyl]-N,N-diethylbenzamide] and the mu agonist analgesics methadone, morphine, and nalbuphine in an assay of capsaicin-induced thermal allodynia in rhesus monkeys. Thermal allodynia was produced by topical application of capsaicin to the tail. Antiallodynic effects of methadone, morphine, and nalbuphine were evaluated alone or in combination with fixed proportions of SNC80 identical to proportions previously shown to enhance acute thermal antinociceptive effects of these mu agonists in rhesus monkeys (0.9 : 1 SNC80/methadone; 0.29 : 1 SNC80/morphine; 3.6 : 1 SNC80/nalbuphine). Methadone, morphine, and nalbuphine each produced dose-dependent antiallodynia. SNC80 produced partial antiallodynia up to the highest dose tested (5.6 mg/kg). SNC80 produced a modest, enantioselective, and naltrindole-reversible enhancement of methadone-induced antiallodynia. However, SNC80 did not enhance morphine antiallodynia and only weakly enhanced nalbuphine antiallodynia. Overall, SNC80 produced modest or no enhancement of the antiallodynic effects of the three mu agonists evaluated. These results suggest that delta agonist-induced enhancement of mu agonist antiallodynia may be weaker and less reliable than previously demonstrated enhancement of mu agonist acute thermal nociception.

## 1. Introduction

Mu-opioid receptor agonists are effective analgesics for the treatment of many types of pain, but their clinical use is limited by undesirable effects that include sedation, respiratory depression, constipation, and high abuse liability [1, 2]. One strategy to improve the effectiveness and/or safety of mu agonist analgesics is to combine them with adjuncts that target other pharmacological systems. For example, we and others have reported that delta opioid receptor agonists can selectively enhance the antinociceptive effects of mu agonists in assays of acute thermal pain while producing a lesser enhancement, no enhancement, or an attenuation of many undesirable effects of mu agonists [3–7]. Moreover, mu agonists may produce a reciprocal attenuation in undesirable effects produced by some delta agonists (e.g., convulsant activity [3]). These findings have been interpreted to suggest that mixtures of delta and mu agonists, or single compounds with mixed agonist activity at both delta and mu receptors, may be useful as alternatives to selective mu agonists for the treatment of pain.

The majority of studies on mu/delta antinociceptive interactions have been conducted in assays that evaluate behavioral responses to acute noxious stimuli, which are defined as stimuli capable of producing tissue damage. However, responses to noxious stimuli are adaptive (e.g., by promoting movement of an affected limb away from a stimulus that could cause tissue damage), and opioids are used clinically to dampen these responses only as an adjunct to other drugs in the context of general or epidural anesthesia [8–10]. The far more common use of opioid analgesics is to treat spontaneous or hypersensitive pain responses associated with states such as inflammation, neuropathy, or cancer [1, 2]. For example, inflammation consequent to a burn, surgical procedure, or other tissue damage is commonly associated with allodynia, which is defined as a pain-like response to normally innocuous stimuli, and opioid analgesics reliably attenuate inflammation-associated allodynia [11, 12]. Preclinical procedures have been developed to examine effects of opioids and other drugs on inflammation-associated allodynia [13], but interactions between delta and mu agonists in these clinically relevant procedures have not been evaluated.

To address this knowledge gap, the present study evaluated interactions between the nonpeptidic delta agonist SNC80 and the mu-opioid agonists methadone, morphine, and nalbuphine in an assay of capsaicin-induced thermal allodynia in rhesus monkeys. Capsaicin is an agonist at TRPV1 receptors (Transient Receptor Potential cation channel, subfamily V, member 1), which are densely and selectively localized on the peripheral terminals of small unmyelinated C-fiber nociceptors [14]. Capsaicin-induced stimulation of TRPV1 receptors increases excitability of these nociceptors and produces an inflammation-like hypersensitivity to thermal stimuli [15]. Either mu or delta agonists administered alone have been shown previously to produce antiallodynic effects in this procedure [16, 17]. In this study, SNC80 and each mu agonist were administered as fixed-proportion mixtures that were shown previously to produce synergistic effects in an assay of acute thermal nociception in rhesus monkeys [6]. We hypothesized that these same proportions of SNC80 would enhance the antiallodynic effects of the mu agonists.

## 2. Materials and Methods

### 2.1. Subjects

Three male and one female adult rhesus monkeys (*Macaca mulatta*; Covance, Denver, PA) weighed between 7 and 10 kg during the course of these studies. All monkeys had prior exposure to drugs (primarily dopaminergic and opioid compounds) and to the behavioral procedures in which they were tested. The subjects were individually housed, and water was freely available. Their diet consisted of Purina Lab Diet Fiber-Plus Monkey Biscuits no.5049 (PMI Feeds, Inc., St. Louis, MO) supplemented with fresh fruit twice weekly. A 12 h light/12 h dark cycle was in effect (lights on from 7 AM–7 PM). Animal maintenance and research were conducted in accordance with the guidelines provided by the NIH Committee on Laboratory Animal Resources. The facility was licensed by the United States Department of Agriculture and accredited by the Association for the Assessment and Accreditation of Laboratory Animal Care. Protocols were approved by the Institutional Animal Care and Use Committee. The health of the monkeys was monitored daily by technical and veterinary staff. Monkeys had visual, auditory, and olfactory contact with other monkeys throughout the study. Monkeys also had access to puzzle feeders, mirrors, and chew toys to provide environmental enrichment. Music was played daily in all housing rooms.

### 2.2. Assay of Capsaicin-Induced Thermal Allodynia

Monkeys were seated in acrylic restraint chairs so that their tails hung down freely. To determine tail-withdrawal latencies, the lower 15 cm of each monkeys shaved tail was immersed into a thermal container of warm water heated to the designated temperature (see below for temperatures). The latency in seconds for the monkey to remove its tail from the water was measured using a handheld stopwatch. If the subject did not withdraw its tail within 20 s, the tail was removed from the water by the experimenter, and a latency of 20 s was assigned to that measurement.

Experimental sessions were conducted once per week. At the beginning of each session, tail withdrawal latencies were determined for each monkey from water heated to 38, 42, 46, and 50°C, and the order of temperature presentations was randomized across sessions. By this procedure, baseline temperature-effect curves were determined in each monkey at the beginning of each session, and the highest temperature that failed to elicit tail withdrawal was determined (i.e., the highest temperature to produce a tail withdrawal latency of 20 sec). Water heated to this “threshold” temperature then served as the thermal stimulus for subsequent studies of allodynia during that session. The threshold stimulus intensity was 42°C for two monkeys and 46°C for the other two monkeys throughout the study.

Allodynia was elicited by topical application of capsaicin as described previously [15, 18]. Following baseline tail withdrawal latency determinations, a topical patch treated with capsaicin solution or vehicle was prepared as described below (see Section 2.4), and the patch was applied to a region approximately 7 cm from the bottom of the tail for 5 min. After 5 min, the patch was removed. Tail withdrawal latencies were then redetermined 15, 30, 45, and 60 min after patch removal using the thermal stimulus identified from the baseline temperature-effect curve in each monkey (i.e., 42°C in two monkeys, 46°C in the other two monkeys).

Three sets of pharmacological manipulations were implemented. (1) First, effects of mu and delta opioid agonists administered alone were determined. Drugs and doses studied were the high-efficacy mu agonist methadone (0.32–3.2 mg/kg), the intermediate-efficacy mu agonist morphine (0.1–1.0 mg/kg), the low-efficacy mu agonist nalbuphine (0.032–0.32 mg/kg), the high-efficacy delta agonist SNC80 (1.0–5.6 mg/kg), and the inactive enantiomer of SNC80, SNC67 (5.6 mg/kg). (2) Second, effects of delta/mu drug mixtures were studied. The dose proportions of SNC80 in combination with methadone (0.9 : 1 SNC80/methadone), morphine (0.3 : 1 SNC80/morphine) and nalbuphine (3.6 : 1 SNC80/nalbuphine) were based on a previous study that examined effects of these mixtures in other behavioral procedures in monkeys [6]. Specifically, these SNC80/mu agonist proportions produced selective and synergistic enhancement of the thermal antinociceptive effects of methadone, morphine, and nalbuphine. The proportion of SNC67 in combination with methadone (0.9 : 1) was designed to match the SNC80/methadone mixture with the highest proportion of SNC80. (3) Third, antagonism studies were conducted to evaluate the role of delta and mu receptors in mediation of effects produced by methadone alone and by the 0.9 : 1 SNC80/methadone mixture. Dose-effect curves for methadone and 0.9 : 1 SNC80/methadone were redetermined after pretreatment with the delta-selective antagonist naltrindole (1.0 mg/kg) and the mu-selective opioid antagonist naltrexone (0.1 mg/kg). In all studies, agonists and mixtures were administered 15 min before application of the capsaicin patch, and antagonists were administered 30 min before the agonist or mixture. All studies with methadone and morphine were conducted in one group of 3 monkeys. All studies with nalbuphine were conducted in a different group of 3 monkeys. Two monkeys were used for all studies with all drugs.

### 2.3. Data Analysis

Raw tail withdrawal latencies obtained 15, 30, 45, and 60 min after removal of the capsaicin patch were converted to Percent Maximum Possible Effect (%MPE) using the equation %MPE = [(Test Latency − Capsaicin Alone Latency)/(20 − Capsaicin Alone Latency)] ∗ 100, where “test latency” was the tail withdrawal latency obtained at each time point after drug pretreatment + capsaicin patch treatment, and “Capsaicin Alone Latency” was the latency obtained at the corresponding time point after treatment with the capsaicin patch alone. Data from all four time points were then averaged to yield a mean %MPE for each monkey during each session, and these mean %MPE values were then used for graphs and statistical analysis.

 ED50 values and 95% confidence limits were determined by linear regression as the dose required to produce 50 %MPE (Prism 4.0c for Macintosh; GraphPad Software, San Diego, CA). ED50s were considered to be significantly different if 95% confidence limits did not overlap. Data for SNC80/mu agonist interactions were also analyzed by 2-way analysis of variance (ANOVA), with mu agonist dose as one factor and SNC80 proportion as the second factor. A significant ANOVA was followed by the Holm-Sidak post hoc test. The criterion for significance was *P* < 0.05.

### 2.4. Drugs

Methadone HCl, morphine sulfate, and naltrexone HCl were provided by the Drug Supply Program of the National Institute on Drug Abuse (Bethesda, MD). SNC80, SNC67, nalbuphine HCl, and naltrindole HCl were provided by Kenner C. Rice (NIDA and NIAAA, Bethesda, MD). Methadone, morphine, nalbuphine, naltrexone, and naltrindole were dissolved in sterile water. SNC80 and SNC67 were free bases dissolved in 2-3% lactic acid and sterile water to a final concentration of 50 mg/mL, and dilutions were made with sterile water. All drugs were administered IM in the thigh, and doses are expressed in the salt or base forms given above.

Capsaicin (Sigma Chemical Co., St. Louis, MO) was dissolved in a vehicle composed of 70% alcohol and 30% sterile water, and it was delivered transdermally (topical patch) as described previously [15, 19]. Specifically, the patch was composed of layers consisting of a 1 cm^2^ piece of tissue, 1-cm^2^ piece of two-ply gauze, and a round adhesive bandage affixed to adhesive backing (23 mm wide; Nexcare Active, NM). The patch was then attached to a 12 cm long elastic tape. The capsaicin solution (0.3 mL) was slowly dripped from a syringe onto the patch, and the concentration of capsaicin in the solution was individually determined for each monkey as the lowest concentration to produce sustained decreases in tail-withdrawal latencies from the threshold temperature to ≤5 sec throughout the 1 hr testing period [0.61 mg/mL (2 mM) in three monkeys and 2.44 mg/mL (8 mM) in the fourth monkey]. Within 30 s of preparing the capsaicin patch, it was secured onto the monkey's tail with the elastic tape and left on for 5 min.

## 3. Results

**Figure 1:**
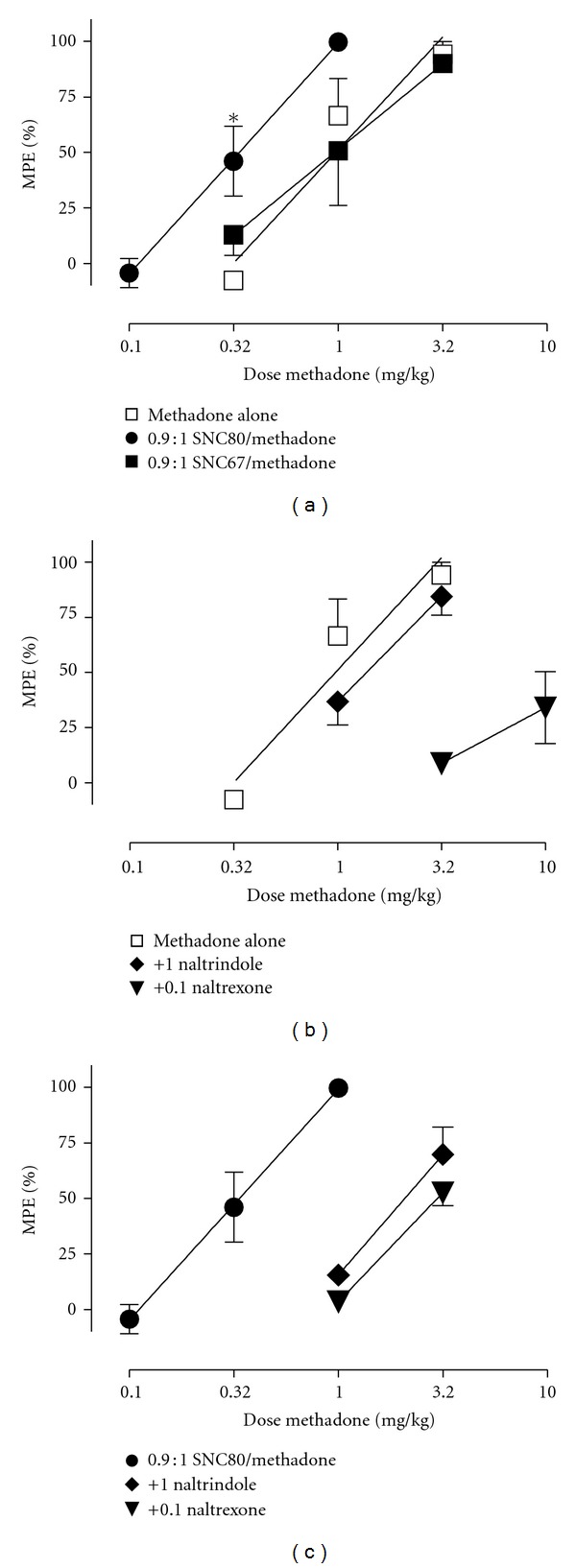
Antiallodynic effects produced by methadone administered alone or in combination with SNC80 or SNC67 (a), methadone after pretreatment with 0.1 mg/kg naltrexone or 1.0 mg/kg naltrindole (b), and 0.9 : 1 SNC80/methadone after pretreatment with naltrexone or naltrindole (c). Abscissae: dose methadone in mg/kg (log scale). Ordinates: percent maximum possible effect (%MPE). All points show mean ± SEM for three monkeys. ED50 values are compared in Table 1. Data for methadone alone and 0.9 : 1 SNC80/methadone at doses of 0.32 and 1.0 mg/kg methadone in the left panel were compared by 2-way ANOVA (see text for details). The asterisk in the left panel indicates a significant difference from methadone alone.

**Figure 2:**
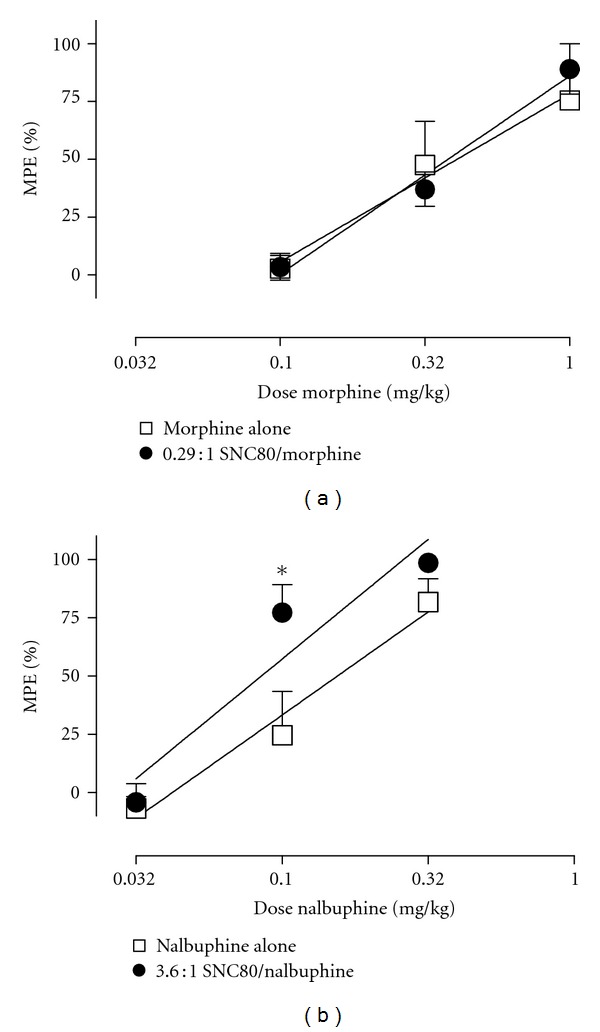
Antiallodynic effects of morphine (a) or nalbuphine (b) administered alone or in a mixture with SNC80. Abscissae: dose morphine or nalbuphine in mg/kg (log scale). Ordinates: percent maximum possible effect (%MPE). All points show mean ± SEM data from 3 monkeys. ED50 values are compared in Table 1, and data in each panel were analyzed by 2-way ANOVA (see text for details). The asterisk in the right panel indicates a significant difference from nalbuphine alone.

The mean baseline tail withdrawal latency (±SEM) from water heated to the threshold temperature was 20.0 ± 0 sec during the course of the study. Treatment with capsaicin alone reduced tail withdrawal latencies to mean ± SEM values of 2.2 ± 0.2 sec. The effects of methadone, morphine and nalbuphine alone are shown in Figures 1 and 2 (open squares). All three mu agonists dose dependently blocked capsaicin-induced thermal allodynia, and ED50 values for each compound are shown in Table 1. SNC80 at doses up to 3.2 mg/kg failed to produce antiallodynia in any monkey (maximal mean ± SEM  %MPE = −5.5 ± 3.6). A higher dose of 5.6 mg/kg SNC80 produced modest antiallodynic effects in two monkeys (%MPE of 56.5 and 37.5%), but this dose produced a convulsion in a third monkey and was not tested further. A dose of 5.6 mg/kg SNC67 had no effect in any monkey (mean ± SEM  %MPE = 7.2 ± 3.1).

Figure 1(a) shows effects of methadone in combination with SNC80 and SNC67. There was a tendency for SNC80 to increase the potency of methadone as indicated by a parallel left shift in the methadone dose-effect curve. The SNC80-induced reduction in the methadone ED50 was not statistically significant (Table 1). However, 2-way ANOVA indicated significant main effects of methadone dose [*F*(1,2) = 27.4, *P* = 0.035] and SNC80 proportion [*F*(1,2) = 157.1, *P* = 0.006], but not a significant interaction [*F*(1,2) = 2.1, *P* = 0.289]. The SNC80/methadone mixture produced significantly greater antiallodynia than methadone alone at a methadone dose of 0.32 mg/kg (*P* < 0.05). SNC67, the inactive enantiomer of SNC80, had no effect on the methadone dose-effect curve or ED50 value. Figures 1(b) and 1(c) and Table 1 also show effects of the mu antagonist naltrexone and the delta antagonist naltrindole on antiallodynia induced by methadone alone or the 0.9 : 1 SNC80/methadone mixture. Naltrexone significantly increased ED50 values for both methadone and the SNC80/methadone mixture, but naltrindole increased the ED50 value only for the mixture.

Figure 2 shows the effects of morphine and nalbuphine in combination with SNC80, and ED50 values are shown in Table 1. These proportions of SNC80 failed to significantly alter the ED50 values for either morphine or nalbuphine. Two-way ANOVA also failed to show a significant effect of SNC80 on morphine-induced antiallodynia. However, 2-way ANOVA did reveal significant main effects of nalbuphine dose [*F*(2,4) = 72.7, *P* < 0.001] and SNC80 proportion [*F*(1,2) = 19.9, *P* = 0.047] as well as a significant interaction [*F*(2,4) = 8,4, *P* = 0.037]. The 3.6 : 1 SNC80/nalbuphine mixture produced significantly greater antiallodynia than nalbuphine alone at a dose of 0.1 mg/kg nalbuphine (*P* < 0.05).

## 4. Discussion

### 4.1. Effects of Mu Agonists and SNC80 Alone

In agreement with previous studies, the opioid analgesics methadone, morphine, and nalbuphine each produced a dose-dependent and complete blockade of capsaicin-induced thermal allodynia in rhesus monkeys [16, 17, 20, 21]. The sensitivity of methadone-induced antiallodynia to antagonism by the mu-selective antagonist naltrexone but not the delta-selective antagonist naltrindole suggests that methadone effects in this procedure were mediated by mu-opioid receptors. Similar data suggest that the antiallodynic effects of morphine are also mu-receptor mediated in this procedure [16]. Receptor mediation of the antiallodynic effects of nalbuphine has not been investigated, but many other effects of nalbuphine in rhesus monkeys are mu-receptor mediated [22, 23]. Methadone, morphine, and nalbuphine are distinguished in part by their different efficacies at mu receptors, with methadone having very high efficacy, morphine intermediate efficacy, and nalbuphine relatively low efficacy [24, 25]. The finding that all three opioids fully blocked capsaicin-induced allodynia suggests that this procedure has relatively low requirements for efficacy at mu-opioid receptors. These data are also consistent with the broad utility of mu agonist analgesics for the treatment of inflammation-associated pain.

Effects of SNC80 and SNC67 administered alone are also consistent with previous studies [16, 26]. Thus, SNC80 at doses up to 5.6 mg/kg produced partial antiallodynia, whereas the inactive enantiomer of SNC80, SNC67 was inactive. The enantioselectivity and naltrindole reversibility [16] of SNC80-induced antiallodynia are consistent with delta-receptor mediation. In previous studies, a higher dose of 10 mg/kg SNC80 completely blocked capsaicin-induced allodynia, and SNC80 also blocked or reversed thermal allodynia produced by prostaglandin E2 and complete Freund's adjuvant [16]. In this study, doses above 5.6 mg/kg SNC80 were not evaluated due to the emergence of convulsant effects in one monkey. These convulsant effects have been described previously, although they are usually produced only at doses ≥10 mg/kg in rhesus monkeys [27, 28]. Overall, these results suggest that delta agonists alone may have value for the treatment of inflammation-associated pain, but the utility of at least some of these compounds may be limited by the risk of convulsant activity.

### 4.2. Effects of SNC80 in Combination with Mu Agonists

Previous studies in rhesus monkeys found that SNC80 and other high-efficacy delta agonists enhanced the acute thermal antinociceptive effects of various mu agonists including methadone, morphine, and nalbuphine [5, 6, 29, 30]. Numerous studies have also been conducted to examine determinants of delta/mu interactions in assays of acute nociception in squirrel monkeys and rodents, and under many circumstances, delta agonists enhanced the antinociceptive effects of mu agonists [4, 7, 31, 32]. The present study extends these evaluations of delta/mu interactions to an assay of inflammation-like thermal allodynia in rhesus monkeys. As in the assay of acute thermal nociception in rhesus monkeys [6], SNC80 in a 0.9 : 1 SNC80/methadone mixture enhanced the antiallodynic effects of methadone. These effects were enantioselective and naltrindole reversible, suggesting that SNC80-induced enhancement of methadone antiallodynia was mediated by delta opioid receptors. However, by at least two measures, SNC80-induced enhancement of mu agonist antiallodynia was weaker and less consistent than SNC80-induced enhancement of mu agonist acute thermal antinociception. First, SNC80 produced a nonsignificant and less than 3-fold decrease in the mean ED50 value for methadone-induced antiallodynia. A significant effect of SNC80 was revealed only by two-way ANOVA at a single methadone dose. In contrast, the same proportion of SNC80 produced a significant and more than a 5-fold decrease in the ED50 value for methadone-induced acute thermal antinociception. Second, proportions of SNC80 that significantly enhanced the acute thermal antinociceptive effects of the lower-efficacy mu agonists morphine and nalbuphine produced little or no enhancement of the antiallodynic effects of these compounds in the present study.

There are several potential explanations for the relatively modest delta/mu interactions observed in this study. For example, it is well established that one critical determinant of drug interactions is the proportion of drugs in a mixture [33]. This study employed proportions of SNC80 that had been shown previously to produce a robust and synergistic enhancement of the acute thermal antinociceptive effects of methadone, morphine, and nalbuphine in rhesus monkeys [6]. Although these proportions of SNC80 were less effective in enhancing antiallodynic effects of these mu agonists, it is possible that other delta agonist proportions might be more effective. At the very least, though, the present data suggest a dissociation between optimal proportions of SNC80 to enhance acute thermal antinociceptive versus antiallodynic effects of mu agonists. Another possibility is that mu agonist-induced antinociception and antiallodynia may rely on different neural substrates with different capabilities for supporting synergistic delta/mu interactions. Thus, mu agonist-induced acute thermal antinociception requires activity at mu receptors located in the central nervous system, whereas mu antiallodynia may also be mediated by peripheral mu receptors located outside the central nervous system (e.g., on peripheral terminals of nociceptors) [17, 21]. In accordance with effects of SNC80/methadone mixtures in this study, results with the mixed-action peptidic mu/delta agonist MMP2200 [H(2)N-Tyr-D-Thr-Gly-Phe-Leu-Ser-(O-beta-D-lactose)-CONH(2)] have provided evidence to suggest that peripheral mu and delta receptors can interact synergistically to mediate antiallodynia in rhesus monkeys [18]. However, the degree of delta/mu synergy may be less for peripheral versus central opioid receptor populations.

### 4.3. Clinical Implications

Insofar as this assay of capsaicin-induced thermal allodynia models a clinically relevant dimension of inflammatory pain, the present results suggest that SNC80 and other delta agonists may have limited impact on mu agonist analgesia in the context of inflammation. However, even modest degrees of enhancement may be beneficial if mixtures result in attenuated side effects, and previous studies do suggest that mixtures of delta and mu agonists may be less potent or less effective than delta or mu agonists alone in producing such undesirable effects as suppression of food-maintained operant responding, reinforcing effects, convulsions, or respiratory depression [3, 5, 6, 34]. Moreover, the present study modeled only one dimension (thermal allodynia) of inflammatory pain. It is possible that other dimensions of pain (e.g., mechanical allodynia or spontaneous pain) may be more sensitive to delta/mu interactions. For example, although this is the first study to assess delta/mu interactions in an assay of inflammation-like thermal hypersensitivity, synergistic delta/mu interactions have been observed in mitigating mechanical antiallodynia caused in rats by spinal nerve ligation [35] or intraplantar administration of nerve growth factor [36]. Overall, the range of conditions under which delta/mu interactions may increase the effectiveness and/safety of mu agonist analgesics would benefit from further study.

## 5. Conclusions

The main finding of this study was that the delta agonist SNC80 produced only a modest enhancement of mu agonist-induced antiallodynia in rhesus monkeys. These findings contrast with the more consistent enhancement by SNC80 and other delta agonists of mu agonist-induced acute thermal antinociception in rhesus monkeys. These results suggest that delta agonists may have only limited utility in enhancing analgesic effects of mu agonists in the clinically relevant context of inflammatory pain.

## Figures and Tables

**Table 1:** ED50 values (95% confidence limits) in mg/kg for the antiallodynic effects of methadone, morphine, and nalbuphine administered alone or in combination with other drugs. Symbols indicate significantly different from mu agonist alone (*) or from 0.9 : 1 SNC80/methadone alone (†) as indicated by nonoverlapping confidence limits.

Treatment	ED50 (95%CL)
Methadone	
Methadone alone	0.98 (0.48–1.97)
0.9 : 1 SNC80/methadone	0.34 (0.21–0.56)
0.9 : 1 SNC67/methadone	1.03 (0.27–3.31)
Methadone + 0.1 NTX	>10*
Methadone + 1.0 NTI	1.37 (0.22–3.61)
0.9 : 1 SNC80 methadone + 0.1 NTX	2.97 (2.09–4.86)^†^
0.9 : 1 SNC80/methadone + 1.0 NTI	2.08 (0.98–5.52)^†^

Morphine	
Morphine alone	0.41 (0.14–1.60)
0.29 : 1 SNC80/morphine	0.38 (0.22–0.68)

Nalbuphine	
Nalbuphine alone	0.15 (0.07–0.43)
3.6 : 1 SNC80/nalbuphine	0.08 (0.04–0.17)
